# Supplementary material for: Targeted Bioluminescent Imaging of Pancreatic Ductal Adenocarcinoma Using Nanocarrier-Complexed EGFR-Binding Affibody–Gaussia Luciferase Fusion Protein
Source: Pharmaceutics. 2023 Jul 19;15(7):1976. doi: 10.3390/pharmaceutics15071976 (PMC10384630; doi:10.3390/pharmaceutics15071976)
Supplement: Supplementary file 1 [file pharmaceutics-15-01976-s001.zip › pharmaceutics-2501044-supplementary.pdf]

Supplementary Materials: Targeted Bioluminescent Imaging of Pancreatic Ductal Adenocarcinoma Using Nanocarrier-Complexed EGFR-Binding Affibody–Gaussia Luciferase Fusion Protein

Jessica Hersh, Yu-Ping Yang, Evan Roberts, Daniel Bilbao, Wensi Tao, Alan Pollack, Sylvia Daunert and Sapna K. Deo

Supplementary Table S1. Best-fit values for the kinetic curves in Figure 2F. A two phase decay nonlinear regression model was fit to each curve, and the best-fit values of unshared parameters were compared between the data sets. All parameters were compared to determine whether one curve adequately fits all data sets. Using Extra sum-of-squares F Test, it was determined that $p<0.0001$ and the curves differ between data sets.

Best-fit values	ZEGFR-GLuc	ZEGFR-GLuc-PAMAM
Y0	113.2	113.1
Plateau	4.627	2.962
PercentFast	67.19	60.57
KFast	0.06982	0.05947
KSlow	0.005221	0.003953

Figure S1. EGFR-positivity

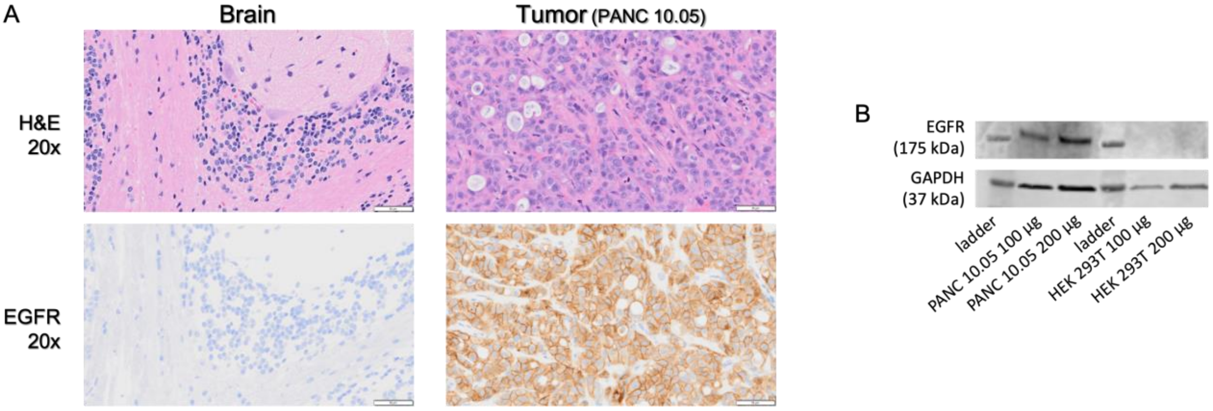

Figure S1. EGFR-positivity. EGFR-positivity was confirmed in both the in vivo xenograft mice tumors and in vitro in the PANC 10.05 cell line. (**a**) Tissue staining for EGFR was done in slices from the brain and from the tumor, and showed that EGFR was found in the tumor cells. (**b**) Western blotting probing for EGFR was performed for PANC 10.05 cells and HEK 293T cells as a negative control to demonstrate EGFR-positivity.

Figure S2. Signal at 3 h and 6 h

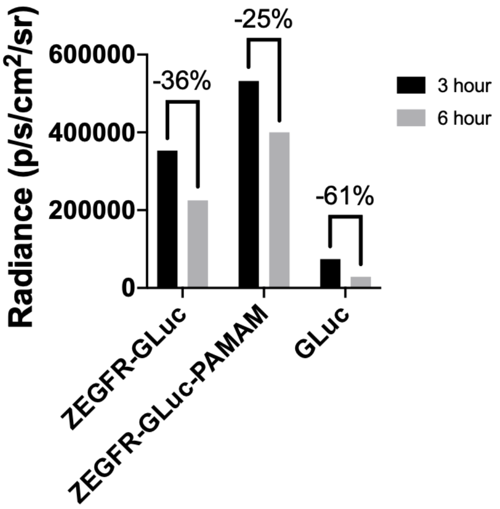

Figure S2. Signal at 3 h and 6 h. Signal decrease between the radiance at 3 h post-treatment and 6 h post-treatment. The graph shows quantification of the radiance for the representative mice images in Figure 4A. The signal decreased less (25% decrease) with the targeted complex compared to the fusion protein alone (36% decrease).

Figure S3. Additional capabilities of the fusion protein and targeted complex for *in vivo* imaging

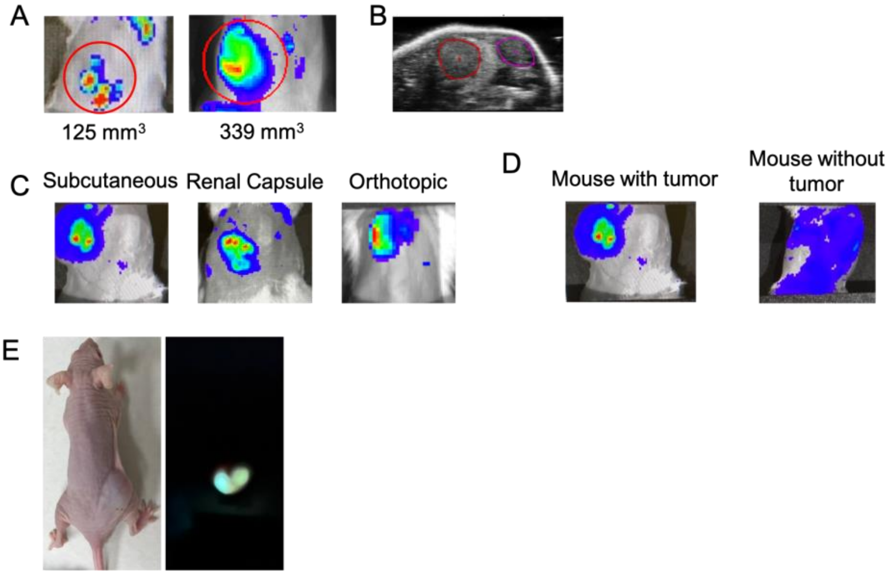

Figure S3. Additional capabilities of the fusion protein and targeted complex for *in vivo* imaging. (A) IVIS bioluminescent image showing the targeted complex identified two separate masses (left side) and can localize to various sizes of tumor. (B) Ultrasound imaging confirming the presence of two individual masses. The masses are indicated by the red and pink circles. (C) IVIS images of the targeted complexes in multiple mouse models, including subcutaneous, renal capsule, and orthotopic xenograft mice. (D) When a tumor is present (left mouse), the targeted complex localizes to the tumor. In the absence of a tumor (right mouse), there is low background signal throughout the mouse. (E) A nude mouse with a subcutaneous xenograft was injected with the fusion protein. Images were taken with a cell phone with the lights on (left side) and off (right side). The bioluminescent intensity is strong enough to be seen without the use of imaging equipment, such as IVIS, for detecting bioluminescent signal in mice.

Sequence S1. ZEGFR-GLuc fusion protein amino acid sequence

The fusion protein was developed by encoding the sequence for the EGFR-targeting affibody, ZEGFR, and the sequence encoding GLuc, into a plasmid (Figure 2A). A linker composed of serine and glycine was used to separate the two proteins in the resulting fusion protein. The amino acid sequence is written in its entirety below. The sequence before the serine-glycine linker encodes the ZEGFR protein, and the sequence after the serine-glycine linker encodes the GLuc.

GVDNKFNKEMWAAWEEIRNLPNLNGWQMTAFIASLVDDPSQSANLLAEAKKLNDAQAPKSSSSG
SSSSGSSSSGMKPTENNEDFNIVAVASNFATTDLDADRGKLPGKKLPLEVLKEIEANARKAGCTRG
CLICLSHIKCTPKMKKWLPGRCHTYEGDKESAQGGIGEAIVDIPEIPGFKDLEPIEQFIAQVDLCVDC
TTGCLKGLANVQCSDLLKKWLPQRCATFASKIQGQVDKIKGAGGD

Methods S1. Western blotting

Western blotting was performed following standard protocols. Briefly, protein collected from cell lysates were mixed at a 1:1 ratio with 2X Laemmli Sample Buffer loading dye (prepared according to manufacturer's instructions: 950 μL Laemmli sample buffer, Bio-Rad Laboratories, Inc, Hercules, CA, USA, combined with 50 μL with 2-Mercaptoethanol, Sigma-Aldrich, St. Louis, MO, USA) and heated at 90 °C for 10 min. The proteins were then separated using a 4-20% mini-PROTEAN TGX precast gel (Bio-Rad Laboratories, Inc, Hercules, CA, USA), and transferred to a 0.2 μm PVDF membrane using Trans-Blot Turbo Transfer Pack (Bio-Rad Laboratories, Inc, Hercules, CA, USA) for western blot analysis. The EGFR and GAPDH primary antibodies (Cell Signaling Technology, Danvers, MA, USA) were incubated overnight at 4 °C and the IRdye® 800CW goat anti-mouse secondary antibody (LI-COR, Lincoln, NE, USA) was incubated for 2 h at room temperature. The membrane was probed for EGFRs and for GAPDH as a control band, and imaged with LI-COR Odyssey CLx western blot imaging system (LI-COR, Lincoln, NE, USA).
